# STAT trial: stoma or intestinal anastomosis for necrotizing enterocolitis: a multicentre randomized controlled trial

**DOI:** 10.1007/s00383-024-05853-3

**Published:** 2024-10-29

**Authors:** Simon Eaton, Niloofar Ganji, Mandela Thyoka, Maher Shahroor, Augusto Zani, Hazel Pleasants-Terashita, Ali El Ghazzaoui, Jayaram Sivaraj, Stavros Loukogeorgakis, Paolo De Coppi, Sandra Montedonico, Sanja Sindjic-Antunovic, Marija Lukac, James Hamill, Candy Suet Cheng Choo, Shireen Anne Nah, Jan Hulscher, Sherif Emil, Aigars Petersen, Rene Wijnen, Cornelius Sloots, David Sigalet, Edward Kiely, Jan F. Svensson, Tomas Wester, Agostino Pierro

**Affiliations:** 1 UCL Great Ormond Street Institute of Child Health, London, UK; 2 Institute of Medical Sciences, University of Toronto, Toronto, ON Canada; 3 The Hospital for Sick Children, Toronto, ON Canada; 4 Universidad de Valparaíso, Valparaiso, Chile; 5 University of Belgrade, Belgrade, Serbia; 6 Starship Children’s Hospital, Auckland, New Zealand; 7 KK Women’s and Children’s Hospital, Singapore, Singapore; 8 Beatrix Children’s Hospital, Groningen, The Netherlands; 9 Montreal Children’s Hospital, Montreal, Canada; 10 Children’s University Hospital, Riga, Latvia; 11 Sophia Children’s Hospital, Rotterdam, The Netherlands; 12 Alberta Children’s Hospital, Calgary, AB Canada; 13 Great Ormond Street Hospital, London, UK; 14 Karolinska University Hospital, Stockholm, Sweden; 15 Division of General & Thoracic Surgery, The Hospital for Sick Children, Toronto, ON Canada

**Keywords:** Necrotizing enterocolitis, Resection, Anastomosis, Stoma, Parenteral nutrition, Mortality

## Abstract

**Purpose:**

The STAT trial is a multicenter randomized controlled trial in 12 centers worldwide aiming to determine the most effective operation for neonates with necrotizing enterocolitis (NEC) requiring intestinal resection: stoma formation (ST) or primary anastomosis (PA).

**Methods:**

Infants having a primary laparotomy for NEC were randomized intraoperatively to PA or ST if the operating surgeon thought that both were viable treatment options for that patient. The primary outcome (duration of parenteral nutrition [PN]) was evaluated by Cox regression.

**Results:**

Eighty patients were recruited from 2010 to 2019. Infants undergoing anastomosis finished PN significantly earlier than patients undergoing stoma (hazard ratio PA vs. ST 2.38, 95% CI 1.36–4.12 *p* = 0.004). There was no difference in mortality between the two groups (PA 4/35 vs. ST 8/38 *p* = 0.35) or in the rate of complications requiring further unplanned operations (*p* = n.s.). Multiple intestinal complications were more frequent in the stoma group compared to the anastomosis group (ST 12/26 vs. PA 5/31, *p* = 0.02, Fisher’s Exact test).

**Conclusion:**

At laparotomy for NEC, when there is no disease distal to resected intestine, primary anastomosis should be performed as it enhances the recovery from NEC, reduces the risk of multiple intestinal complications and does not increase adverse outcomes.

## Introduction

Many infants with necrotizing enterocolitis (NEC) need laparotomy to resect necrotic bowel, followed by either stoma formation or primary anastomosis. It remains unclear which of these two options is the most effective [1]. Traditionally, enterostomy has been favored when severe inflammation or compromised bowel integrity could negatively impact anastomosis healing and is also considered a safer operation in an acutely ill neonate. The stoma allows the distal bowel to rest and recover before any potential subsequent re-anastomosis. However, despite its use, this approach has notable drawbacks. Enterostomies can pose challenges in maintaining adequate enteral nutrition and weight gain, with high output stomas carrying the risk of significant fluid loss, dehydration and electrolyte imbalances. Additional complications including stenosis, prolapse, and excoriation of the surrounding skin further complicate patient management [2]. Moreover, stoma closure necessitates a second anesthesia and surgical procedure once the patient has stabilized. Extended periods with an enterostomy before intestinal continuity is restored can lead to impaired growth [3, 4].

Retrospective reviews from multiple centers have described resection followed by primary anastomosis as a viable alternative to enterostomy in the treatment of neonates with NEC, including those with multifocal disease, and even extremely low birthweight infants [5, 6]. A meta-analysis comparing enterostomy with primary anastomosis concluded that although mortality was lower following primary anastomosis, the retrospective nature of the included studies meant that primary anastomosis may have been reserved for the less unwell infants, so that the higher mortality following enterostomy may have been due to having been used in sicker infants [7]. Primary anastomosis remains a less-frequently performed procedure than enterostomy formation following resection in NEC, with only 15.8% of infants receiving a resection for advanced NEC having a primary anastomosis, compared with 84.2% having stoma formation across a United States network of Children’s hospitals [8]; interestingly, there is considerable inter-hospital variation, with 50% of hospitals performing only stoma, but 18% of hospitals managing at least 50% of patients with a primary anastomosis. National data from the United Kingdom reflect this, with 23% of patients having a resection for NEC managed with primary anastomosis, 71% of patients with a stoma, and 6% with clip and drop [9].

A randomized controlled trial of primary anastomosis vs. enterostomy formation would ideally be performed to help resolve this issue. However, at laparotomy some infants with NEC may be considered, even by surgeons tending to favor primary anastomosis, to be too unstable or to have too extensive disease to perform a primary anastomosis, whereas some infants with limited resection would be considered to benefit from a primary anastomosis, even by proponents of enterostomy formation for most neonates. We therefore designed a randomized controlled trial in which the decision to randomize is only made when the extent of disease has been assessed at laparotomy, and the operating surgeon feels it safe to perform either procedure, in order to decrease bias. The hypothesis to be tested was that primary anastomosis after intestinal resection offers significant advantages to neonates with NEC including more rapid recovery of the intestine and shorter duration of time on parenteral nutrition, without a higher risk of mortality or major complications.

## Methods

### Trial design and registration

This was an international multicenter randomized controlled trial (RCT). The study was approved by Institute of Child Health/Great Ormond Street Hospital Research Ethics Committee/National Research Ethics Service (09/H0713/58) and each collaborating centre subsequently obtained individual ethical approval. The trial was registered with the International Standard Randomized Controlled Trial Register (ISRCTN): ISRCTN01700960. The principles of the Declaration of Helsinki were followed and parents or guardians of all participants provided written informed consent.

### Study setting

This study was conducted in 12 neonatal surgical intensive care units (NICUs) across Europe, Canada, South America, Singapore, and New Zealand Table 1.

**Table 1:** STAT trial participating centers

Center	Country	Number of patients
The Hospital for Sick Children, Toronto, Ontario	Canada	29
Montreal Children’s Hospital, Montreal	Canada	2
Alberta Children’s Hospital, Calgary, Alberta	Canada	1
Great Ormond Street Hospital, London	United Kingdom	10
University Children’s Hospital, Belgrade	Serbia	19
Astrid Lindgren Children’s Hospital, Stockholm	Sweden	5
Children’s University Hospital, Riga	Latvia	3
Starship Children’s Hospital, Auckland	New Zealand	1
KK Women’s and Children’s Hospital	Singapore	1
Beatrix Children’s Hospital, Groningen	The Netherlands	2
Sophia Children’s Hospital, Rotterdam	The Netherlands	2
Hospital Carlos Van Buren, Valpariso	Chile	4

### Study participants

The *inclusion criteria* for the study were: suspected NEC with need for laparotomy based on radiological signs of intestinal perforation or failure of improvement with medical treatment. The *exclusion criteria* for the trial were: no evidence of NEC (e.g. intestinal volvulus); focal intestinal perforation (since many surgeons would not perform a stoma); extensive NEC precluding intestinal anastomosis (intestinal resection will result in short bowel); NEC affecting the colon that cannot be completely assessed because of risk of bleeding; patient’s instability during the operation, parental refusal of consent.

### Recruitment

The paediatric surgeon approached the families/caregivers of eligible patients to obtain written informed consent for inclusion in the trial, prior to the operation. Enrolment and randomization were completed during the laparotomy when disease findings (presence of NEC) and the extent of the disease were fully assessed. All patients underwent an abdominal exploration through a transverse abdominal incision and were assessed for disease findings and extent of the disease. At this point, only those infants in whom the operating surgeon thought that both operations (intestinal resection with stoma formation or intestinal resection with primary anastomosis) were viable treatment options to that patient (no disease distal to stoma or anastomosis) were enrolled and randomized.

### Randomization

Patients were randomized intra-operatively to (1) stoma formation or (2) primary anastomosis for NEC online by weighted minimization (University of Aberdeen Health Services Research Unit). The minimization criteria used were: (i) weight at enrolment (<1000 g, 1000–2000 g, >2000 g); (ii) mechanical ventilation required (yes, no); (iii) inotropic support required (yes, no); (iv) extent of disease (focal, multifocal); (v) intestine involved (small bowel, large bowel, small and large bowel).

### Intervention

Patients randomly assigned to the stoma group had intestinal resection of non-viable bowel performed and a stoma fashioned at the proximal resection margin. These patients required the stoma to be closed at a later date to establish bowel continuity. Patients randomly assigned to the primary anastomosis group underwent intestinal resection of non-viable bowel and anastomosis of the ends of viable bowel (up to a maximum of two anastomoses depending on the extent of disease).

### Primary and secondary outcomes

The *primary* end point of the study was the duration of parenteral nutrition (days), as this reflects the recovery of intestinal function after NEC and can be affected by complications and/or need for further procedures. The *secondary* end points of the study were: mortality, further unplanned surgical procedures, intestinal complications including stricture (of either anastomosis or remaining intestine, confirmed by a contrast study and/or histology), anastomotic leak, prolapse of stoma, stoma necrosis, intestinal obstruction, high output stoma or recurrence of NEC.

### Sample size estimation

Assuming a standard deviation of 20 days in time to full enteral feeds[10], a power calculation suggested that 66 patients (33 per group) would be sufficient to detect a difference of 14 days in time to full enteral feeding (80% power, *α* = 0.05, two-sided). To account for an estimated mortality rate of approximately 20% in this patient population[10], an additional 14 patients were recruited, resulting in a total of 80 patients (40 per group).

### Data monitoring and interim analysis

Participants were allocated a unique study number, with all study data stored under this number as the identifier and transferred to the coordinating centres (UCL Great Ormond Street Institute of Child Health and Hospital for Sick Children, Toronto) using only this identifier. A Data Monitoring and Ethics Committee was convened to review the data once 40 patients had been recruited.

The criteria for stopping the trial were defined as: (i) a significant difference (*p*<0.01) between the two arms in the duration of parenteral nutrition; or (ii) a significantly (*p*<0.01) greater incidence of mortality; or (iii) a significantly (*p*<0.01) greater incidence of serious complications (intestinal stricture, anastomotic leak, stoma prolapse, stoma necrosis, intestinal obstruction, wound dehiscence, sepsis, intra-abdominal abscess, postoperative intraventricular hemorrhage) in one arm compared to the other, analyzed both as single outcomes and as total cumulative complications.

## Results

### Recruitment

**Fig. 1:**
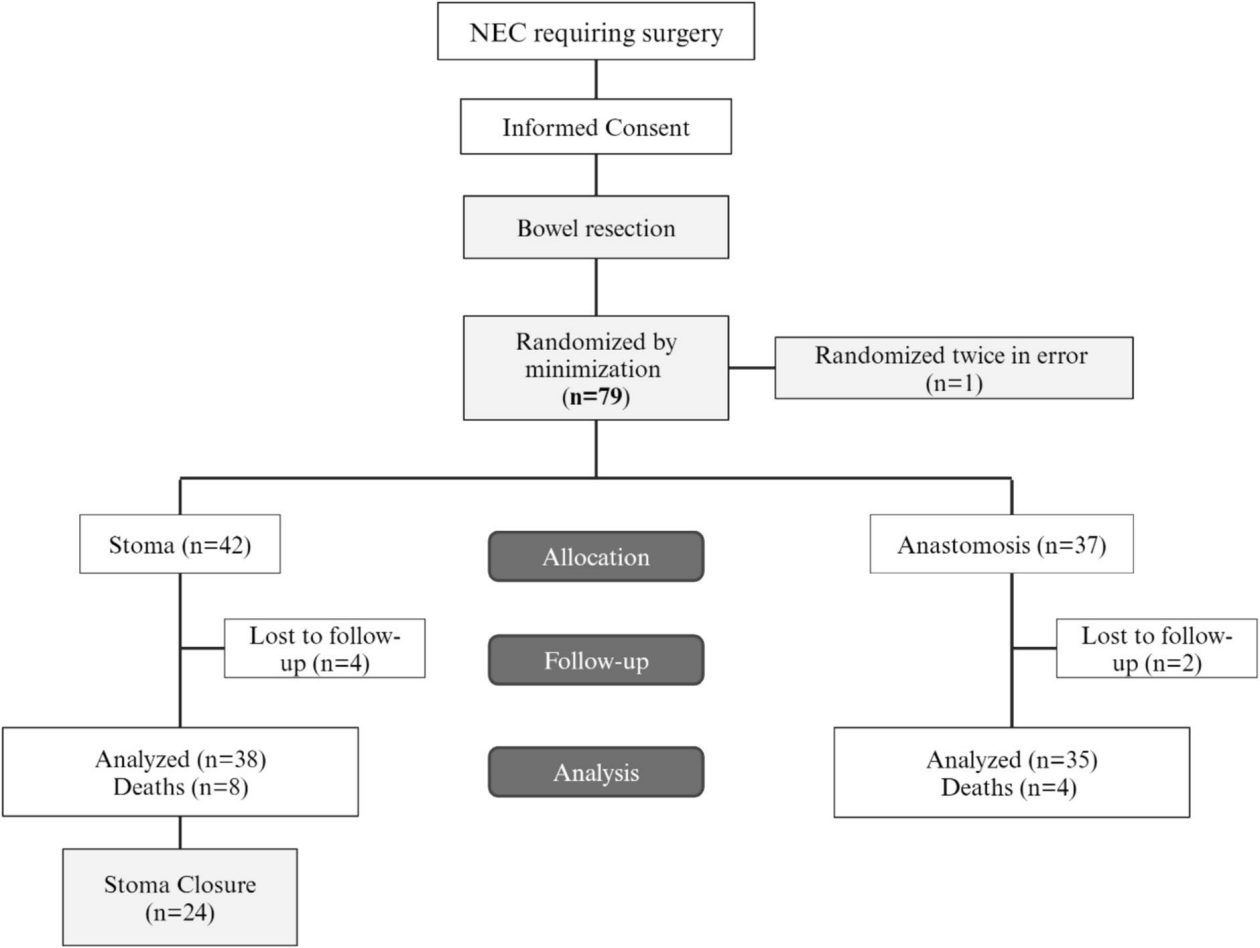
STAT trial flow chart according to Consort guidelines^1^

Eighty patients were recruited between April 2010 and Jan 2019; follow-up at 3 months and 1 year was completed in Jan 2020, recruitment by centre is shown in Table 1. One patient was subsequently determined to have been randomized twice in error leaving 79 patients recruited**.** A Data Monitoring and Ethics Committee was convened in April 2012 and recommended trial continuation. 42 patients were randomized to the stoma group, and 37 patients to the anastomosis group. 6 patients were lost from all follow-ups, including 4 in the stoma group and 2 in the anastomosis group, leaving 38 and 35 patients in each arm, respectively, for analysis Fig. 1.

### Study population

Based on the demographic and clinical characteristics of the study population presented in Table 2, there were no statistically significant differences between the stoma and anastomosis groups across all recorded variables. The mean gestational age was 28.7 weeks in the stoma group and 29.2 weeks in the anastomosis group (*p* = 0.631), while the mean birth weight was 1264 grams and 1319 grams, respectively (*p* = 0.779). The distribution of male infants was similar in both groups (71% in the stoma group vs. 65% in the anastomosis group, *p* = 0.619).

**Table 2:** Patient demographics and clinical characteristics by treatment group*

	Stoma (*n* = 38)	Anastomosis (*n* = 34)	*p* value
Birth characteristics			
Gestational age (weeks)	28.7± 4.7	29.2 ± 4.8	0.631
Birth weight (grams)	1264.2 ± 867	1318.8 ± 771.3	0.779
Male gender (%)	27 (71)	22 (65)	0.619
Admission characteristics			
Age at onset of disease (days)	8.0 (1–51)	7.0 (1–91)	0.702
Age at enrolment (days)	9.5 (2–52)	9.5 (2–97)	0.571
Weight at enrolment (grams)	1392.6 ± 871	1433.6 ± 727	0.832
Mechanical ventilation, n (%)	35 (92)	25 (76)	0.100
Inotrope use, n (%)	11 (29)	10 (29)	1.00
Lost to follow-up at 3 months, n (%)	2 (5)	0	0.494
Lost to follow-up at 1 year, n (%)	3 (8)	0	0.242

Continuous variables are reported as mean ± standard deviation (SD), except for age at onset of disease and age at enrolment which are reported as median (range). Categorical variables are reported as percentages. Comparisons between groups were made using unpaired t-tests for continuous variables and Fisher’s exact test for categorical variables. *p*<0.05 significant.

*Demographics data for one patient from the anastomosis group was missing and is not included in this table.

Regarding admission characteristics, the median age at disease onset and enrollment were comparable between the groups, with a median of 8 days for the stoma group and 7 days for the anastomosis group at disease onset (*p* = 0.702), and 9.5 days at enrollment for both groups (*p* = 0.571). The mean weight at enrollment was also similar (1393 grams in the stoma group and 1434 grams in the anastomosis group, *p* = 0.832). There was a trend towards a higher rate of mechanical ventilation in the stoma group (92% vs. 76%, *p* = 0.100), but this did not reach statistical significance. Inotrope use was equal across both groups (29%, *p* = 1.00). The rate of loss to follow-up at both 3 months and 1 year was minimal and did not differ significantly between the groups.

### Disease characteristics and operation performed

Table 3 presents the intraoperative findings at laparotomy for the stoma and anastomosis groups. The extent of disease was classified as either focal or multifocal. In the stoma group, 51% of patients had focal disease compared to 33% in the anastomosis group, while multifocal disease was observed in 49% of the stoma group versus 67% of the anastomosis group. This difference did not reach statistical significance (*p* = 0.20).

**Table 3:** Findings at laparotomy

	Stoma (*n* = 37*)	Anastomosis (*n* = 33*)	*p* value
Extent of disease			
Focal, n (%)	19/37 (51%)	11/33 (33%)	0.20
Multifocal, n (%)	18/37 (49%)	22/33 (67%)	
Disease location			
Small bowel	25/37 (67%)	19/33(58%)	0.50
Large bowel	5/37 (14%)	8/33 (24%)	
Small and large bowel	7/37 (19%)	6/33 (18%)	

*Data on the extent and location of disease was unavailable for 3 patients, two from the anastomosis group, and one from the stoma group. Data are reported as number (percentage). Comparisons between groups were made using Fisher’s exact test for extent of disease and the Chi-square test for disease location. *p*<0.05 significant

Regarding the location of disease, the involvement of the small bowel alone was slightly more common in the stoma group (67%) than in the anastomosis group (58%). Large bowel involvement was less frequent, observed in 14% of the stoma group compared to 24% in the anastomosis group. The presence of disease in both the small and large bowel was similar between the two groups, at 19% in the stoma group and 18% in the anastomosis group. These differences were not statistically significant (*p* = 0.50). The ileocecal valve was resected in 11/37 patients in the stoma group vs. 11/31 in the primary anastomosis group (*p* = 0.80). Overall, there were no significant disparities in the extent, location of disease or resection of the ileocecal valve between the two treatment groups, suggesting a similar baseline disease burden at the time of surgical intervention.

Of those patients having a stoma, 12 had an end stoma (32%), 1 a loop stoma (3%) and 25 (66%) a divided stoma; one stoma was a jejunostomy, 31 were ileostomies, and 2 were colostomies (location not recorded in 4 patients). Twenty-three patients (74%) of patients having primary anastomosis had a single anastomosis, 8 (26%) had 2 anastomoses (number of anastomoses not recorded in 4 patients).

### Primary outcome (time on parenteral nutrition)

**Fig. 2:**
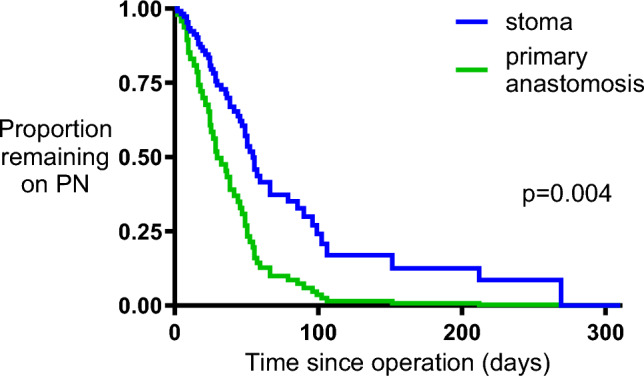
Cox regression analysis of time on parenteral nutrition. Time on parenteral nutrition was compared between groups by Cox regression analysis, adjusting for inotrope use, and censoring for last follow up or death. The graph shows estimated times on parenteral nutrition based on the model fit

Time to end parenteral nutrition was a median of 51 days (range 3–310) in survivors in the stoma group, with 4 patients remaining on PN at the end of follow-up. Time to end parenteral nutrition was significantly shorter (30 [4–105] days) in survivors in the primary anastomosis group (*p* = 0.036 Mann-Whitney test), with one patient remaining on PN at the end of follow-up. These data were also compared by Cox regression analysis, adjusting for the minimization criteria, and censoring for mortality or last follow-up. Randomization group and pre-operative inotropes were the only criteria that remained in the final model, the hazard ratio of finishing PN in the primary anastomosis group compared with stoma was 2.38, 95% CI 1.36–4.16, *p* = 0.004, Fig. 2 whereas the hazard ratio for finishing PN in those receiving inotropes compared with those not receiving inotropes was 0.55 (95% CI 0.29–1.02, *p* = 0.096).

### Mortality

**Fig. 3:**
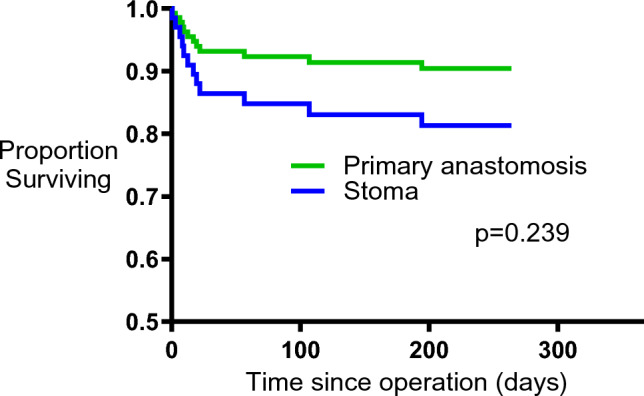
Cox regression analysis of time to death. Survival was compared between groups by Cox regression analysis, adjusting for inotrope use, and censoring for last follow up. The graph shows estimated survival times based on the model fit

There were 12 deaths, 8/38 (21%) in the stoma group and 4/35 (11%) in the primary anastomosis group, *p* = 0.349 (Fisher’s exact test). Survival curves were also compared by Cox regression analysis, adjusting for the minimization criteria and length of follow-up. Pre-operative inotropes and randomization group were the only criteria remaining in the final model. The hazard ratio for mortality in those receiving inotropes compared with those not receiving inotropes was 4.06 (95% CI 1.28–12.82, *p* = 0.017), whereas there was no difference in mortality between stoma and primary anastomosis (hazard ratio for mortality in the stoma group compared with primary anastomosis 2.06 [95% CI 0.62–6.85] *p* = 0.239, Fig. 3).

Causes of death were given in 11/12 patients, and causes of death were listed as multi-system organ failure (*n* = 7), cardiovascular (*n* = 4), sepsis (*n* = 5), NEC (*n* = 5), brain hemorrhage (*n* = 3), prematurity (*n* = 2) [multiple causes of death were listed for some patients]. No mortality could be ascribed as directly resulting from either stoma or primary anastomosis.

### Intestinal complications

Table 4 outlines the intestinal complications observed in both treatment groups. Significantly more patients in the stoma group had multiple intestinal complications compared to the anastomosis group (12/26 vs. 5/31, *p* = 0.02). There was no significant difference in the number of patients with a single complication between the stoma (5/26) and anastomosis groups (6/31, *p* = 1.000). The rates of stricture, wound infection, incisional hernia, wound dehiscence, and leaks were similar between the two groups. As would be expected, stoma-related complications, including stoma necrosis, high stoma output, retracted stoma, and prolapse, were significantly more frequent in the stoma group (15/26) compared to the anastomosis group, which had only one case of stoma necrosis (1/31, *p* = 0.0001, in a patient who had a recurrent anastomotic leak, a perforation and colostomy formation at second laparotomy). The need for additional abdominal operations (excluding stoma closure) did not differ significantly between the groups, with 17/30 patients in the stoma group and 13/31 in the anastomosis group requiring further surgery (*p* = 0.309). Overall, the stoma group had a higher rate of multiple complications and stoma-related issues, indicating a more complex postoperative course compared to the anastomosis group. Stoma closure took place at a median of 77 (30–307) days post-stoma formation.

**Table 4:** Intestinal complications by treatment group (*n* = 60)

	Stoma (*n* = 30)	Anastomosis (*n* = 31)	*p* value
Patients with 1 complication	5/26	6/31	1.000
Patients with >1 complications	12/26	5/31	**0.02**
Stricture	7/30	4/31	0.335
Wound infection	10/26	4/23	0.125
Incisional hernia	3/25	4/24	0.702
Wound dehiscence	7/25	2/23	0.140
Leak	1/30	4/30	0.353
Stoma complications (stoma necrosis, high stoma output, retracted stoma, prolapse of stoma)	15/26	1/31 (stoma necrosis)*	**0.0001**
Additional abdominal operations (excluding stoma closure)	17/30	13/31	0.309

Bold indicates *p* < 0.05 significant

Table excludes deaths (*n* = 12; 4 deaths in the anastomosis group and 8 deaths in the stoma group). Comparisons between groups were made using Fisher’s exact test

*Patient had a recurrent anastomotic leak, a perforation and colostomy formation at second laparotomy

### Medical care

Oxygen was required long term in 13/29 patients in the primary anastomosis group compared with 9/30 patients in the stoma group (*p* = 0.29), and assisted ventilation required long term in 11/29 patients in the primary anastomosis group compared with 9/30 in the stoma group (*p* = 0.589).

## Discussion

In this prospective randomized controlled trial, we have demonstrated that resection with primary anastomosis offers advantages for infants with necrosis and/or perforation due to NEC compared with resection and stoma formation. Infants having a primary anastomosis regained enteral autonomy more rapidly, as judged by time on parenteral nutrition. This was not at a cost of increased mortality. In addition, primary anastomosis decreased the risk of developing intestinal complications.

The principal objectives of the operation in acute NEC are to control sepsis, and to remove gangrenous bowel while preserving as much bowel length as possible. Within these objectives, the two most common operations after intestinal resection are stoma formation or primary anastomosis. The safest surgical option was thought to be resection of non-viable bowel with proximal stoma at the line of resection, to allow fecal diversion and abdominal decompression [11]. Subsequently, several authors have published retrospective case series promoting resection and primary anastomosis in selected cases with good results [5, 6]. Other retrospective studies have compared outcomes following stoma and primary anastomosis, and systematic review and meta-analysis of 12 of these comparative studies has been performed [7]. This meta-analysis concluded that neonates undergoing primary anastomosis were at lower risk of mortality, but suggested that this might be due to a bias of performing enterostomy in sicker babies. The authors also concluded that “*without a sufficiently powered randomized controlled trial, no suggestion can be definitively made regarding the choice of one operative strategy over another*”. As a result of this uncertainty, and lack of even high-quality prospective studies, let alone RCTs, enterostomy formation appears to be still by far the most common procedure performed following a laparotomy for NEC in the US [8] and in the UK [9].

The decision of which procedure to perform can realistically only be made at laparotomy, when the extent of necrosis and the viability of remaining bowel can be assessed. Some infants may have disease that is so extensive that a primary anastomosis cannot be considered, and in some infants a full assessment of distal bowel is impossible, also precluding primary anastomosis. Conversely, some infants may have a limited focal necrotic segment in which almost all surgeons would perform a primary anastomosis. In designing the current trial, it was felt that randomizing patients to stoma vs. primary anastomosis before laparotomy would lead to a major bias, with surgeons more likely to withdraw patients with extensive disease who had been assigned to primary anastomosis, whilst retaining similar sicker patients assigned to the stoma arm. Therefore, a decision was made to randomize at laparotomy when bowel assessment could be performed, and the operating surgeon could establish whether both primary anastomosis and enterostomy were feasible surgical options. Patients were randomized using minimization for the criteria though to be most likely to affect recovery of intestinal function, complications and mortality, namely prematurity (weight at time of operation), patient systemic stability (ventilation, inotropes), and disease severity (extent and location of necrosis). The groups were well balanced, with no significant differences in demographic or minimization criteria.

As patients having a stoma need a second procedure to reverse the stoma, it might be anticipated that ending parenteral nutrition and recovery of enteral function would be faster in a patient with uncomplicated recovery following primary anastomosis than following stoma. However, time on parenteral nutrition was chosen as a primary endpoint, as it was felt to be sensitive to potential complications experienced as a result of primary anastomosis and early return to intestinal continuity (e.g. anastomotic leak, recurrence of NEC, etc.). In the trial, we demonstrated that patients having primary anastomosis had a full return to enteral autonomy 21 days earlier than those having a stoma. There was no significant difference in mortality between the two groups, although there was a trend to lower mortality in the primary anastomosis group. The burden of complications is as would be expected in each group in a population of infants.

Weaknesses of the study include lack of information about patients who were assessed for inclusion but were at laparotomy decided not to be eligible. Nevertheless, the two groups were similar at trial entry, supporting the rationale for intra-operative randomization. The demographic characteristics also reflect the expected population of interest, supporting the relevance of results from the trial. In addition, there were no specific protocols for feeding advancement of for timing of stoma closure. This may have led to a longer time on PN than necessary for the stoma group. From this perspective, the trial can be seen as a pragmatic trial, mirroring current practice of refeeding and timing of stoma closure in NEC infants. There is current interest in shortening the time to stoma closure [12–14], so that stoma reversal in a shorter time than the 77 days observed in the current study may well be safely achievable. Regrettably, we did not collect data on growth of these infants, and so although it is anticipated that an early return to intestinal continuity and ending of parenteral nutrition would lead to improved growth in comparison with those having a stoma, in whom impaired growth is common [3, 4], this remains unproven.

## Conclusions

Primary anastomosis following resection of necrotic and/or perforated bowel in NEC is associated with an earlier end to parenteral feeding than stoma formation, reduced risk of intestinal complications with no increased risk of mortality. Primary anastomosis is the procedure of choice at laparotomy for NEC, when there is no evidence of NEC distal to anastomosis and should be considered in patients of all weights, requiring mechanical ventilation or inotropic support, with focal or multifocal disease involving any part of the small and/or large intestine.

## Data Availability

All data supporting the findings of this study are available within the paper and its Supplementary Information.

## References

[1] Hall NJ, Eaton S, Pierro A (2013) Royal Australasia of Surgeons Guest Lecture. Necrotizing enterocolitis: prevention, treatment, and outcome. J Pediatr Surg 48:2359–67. 10.1016/j.jpedsurg.2013.08.006 24314171 10.1016/j.jpedsurg.2013.08.006

[2] Mutanen A, Pierro A, Zani A (2018) Perioperative Complications Following Surgery for Necrotizing Enterocolitis. Eur J Pediatr Surg 28:148–151. 10.1055/s-0038-1636943 29534255 10.1055/s-0038-1636943

[3] Chong C, van Druten J, Briars G, Eaton S, Clarke P, Tsang T, Yardley I (2019) Neonates living with enterostomy following necrotising enterocolitis are at high risk of becoming severely underweight. Eur J Pediatr 178:1875–1881. 10.1007/s00431-019-03440-6 31522315 10.1007/s00431-019-03440-6 PMC6892362

[4] Davidson JR, Omran K, Chong CKL, Eaton S, Edwards AD, Yardley IE (2024) Exploring Growth Failure in Neonates With Enterostomy. J Pediatr Surg 59:211–215. 10.1016/j.jpedsurg.2023.10.010 37940463 10.1016/j.jpedsurg.2023.10.010

[5] Ade-Ajayi N, Kiely E, Drake D, Wheeler R, Spitz L (1996) Resection and primary anastomosis in necrotizing enterocolitis. J R Soc Med 89:385–8. 10.1177/014107689608900708 8774536 10.1177/014107689608900708 PMC1295852

[6] Hall NJ, Curry J, Drake DP, Spitz L, Kiely EM, Pierro A (2005) Resection and primary anastomosis is a valid surgical option for infants with necrotizing enterocolitis who weigh less than 1000 g. Arch. Surg. 140:1149–1151 16365234 10.1001/archsurg.140.12.1149

[7] Haricharan RN, Gallimore JP, Nasr A (2017) Primary anastomosis or ostomy in necrotizing enterocolitis? Pediatr Surg Int 33:1139–1145. 10.1007/s00383-017-4126-z 28770340 10.1007/s00383-017-4126-z

[8] Goldfarb M, Choi PM, Gollin G (2024) Primary Anastomosis Versus Stoma for Surgical Necrotizing Enterocolitis in US Children’s Hospitals. J Surg Res 295:296–301. 10.1016/j.jss.2023.11.005 38056356 10.1016/j.jss.2023.11.005

[9] British Association of Paediatric Surgeons Congenital Anomalies Surveillance System Necrotising Enterocolitis C, Allin B, Long A-M, Gupta A, Knight M, Lakhoo K, Kazmierski M, Kenny S, Lopes J, Cusick E, Parsons G, Mccabe A, Upadhyaya M, Walker G, De Coppi P, Besarovic S, Thakkar H, Tullie L, Sutcliffe J, Eradi B, Ross A, Maphango N, Motiwale S, Salloum A, Pardy C, Waly R, Charlesworth P, Craigie R, Lall A, Lindley R, Johal N, Njere I, Mortell A, Nandi B, Jones A, Fouad D, Tan Y-W, Kufeji D, Stanwell J, Lakshminarayanan B, Burge D, Wetherill C, Niyogi A, Parsons C, Doyle M, Turner A, Yardley I, Shrestha R, Mullassery D, Paramalingham S, Ragazzi S (2017) A UK wide cohort study describing management and outcomes for infants with surgical Necrotising Enterocolitis. Sci Rep 7:41149. 10.1038/srep41149 28128283 10.1038/srep41149 PMC5269581

[10] Hofman FN, Bax NM, van der Zee DC, Kramer WL (2004) Surgery for necrotising enterocolitis: primary anastomosis or enterostomy? Pediatr Surg Int 20:481–3. 10.1007/s00383-004-1207-6 15197565 10.1007/s00383-004-1207-6

[11] Martin LW, Neblett WW (1981) Early operation with intestinal diversion for necrotizing enterocolitis. J Pediatr Surg 16:252–5. 10.1016/s0022-3468(81)80674-4 7252725 10.1016/s0022-3468(81)80674-4

[12] Dantes G, Murfee J, Doll A, Weaver K, Alemayehu H (2024) Weight at Ostomy Takedown as a Factor to Consider for Operative Timing-Is It Relevant? J Laparoendoscop Adv Surg Tech. 10.1089/lap.2024.0188 10.1089/lap.2024.0188 39162564

[13] Singhal G, Ramakrishnan R, Goldacre R, Battersby C, Hall NJ, Gale C, Knight M, Lansdale N (2024) UK neonatal stoma practice: a population study. Arch Dis Childhood-Fetal Neon Edit. 10.1136/archdischild-2024-327020 10.1136/archdischild-2024-327020 PMC11671890 38897635

[14] Zani A, Lauriti G, Li Q, Pierro A (2017) The Timing of Stoma Closure in Infants with Necrotizing Enterocolitis: A Systematic Review and Meta-Analysis. Eur J Pediatr Surg 27:7–11. 10.1055/s-0036-1587333 27522125 10.1055/s-0036-1587333

